# Health impacts and medical assistance after Libyan flood disaster: Emergency medical teams’ responses

**DOI:** 10.55730/1300-0144.6025

**Published:** 2025-05-19

**Authors:** Hakan GÜNER, Okan MADEN, Muhammed Saltuk DENİZ, Kerem Dost BİLMEZ, Şükrü YORULMAZ, Mehmet Enes GÖKLER

**Affiliations:** 1 Ministry of Health, Republic of Türkiye, Ankara, Turkiye; 2 Department of Public Health, Faculty of Medicine, Ankara Yıldırım Beyazıt University, Ankara, Turkiye

**Keywords:** Emergency medical team, Libya, disaster, flood

## Abstract

**Background/aim:**

Floods are the most frequent natural disasters and pose direct and indirect health risks, some well-documented and others poorly understood. Epidemiological studies help bridge these gaps and guide effective public health responses.

In September 2023, Storm Daniel caused severe flooding in Libya, damaging infrastructure, including healthcare facilities. The disaster affected 250,000 people, displaced 48,000, and claimed 15,000 lives, making it the second deadliest natural disaster of the year. This study aims to assess the characteristics of flood-affected patients to improve disaster preparedness.

**Materials and methods:**

This descriptive study examined disaster victims who visited three clinics established as part of an international aid initiative after the flood disaster in Libya. Data were collected for visits made between September 13, 2023, and November 24, 2023, and categorized by sex, age group, day of visit, and reason for visit.

**Results:**

A total of 5786 clinic visits were recorded between September 13 and November 24, 2023. Among them, 75.3% were male, and the majority of patients (77.0%) were aged 18–64 years. Most visits (69.8%) occurred within 4–30 days’ postdisaster. Acute illnesses and symptoms were the most common reason for visit (55.8%), followed by injuries (22.0%), exacerbations of chronic diseases (13.9%), routine follow-ups (7.9%), and mental health issues (0.4%). The most prevalent subcategory was abrasion/laceration/cuts (18.8%), followed by pain (18.7%) and acute respiratory conditions (17.7%).

**Conclusion:**

This study highlights key findings regarding the healthcare needs of disaster victims following the flood disaster in Libya. The health effects of disasters are influenced by various factors, including sociocultural dynamics. Research into disaster-related health impacts can inform and enhance disaster prevention and management strategies, contributing to improved public health resilience.

## 1. Introduction

Floods, the most common disasters triggered by natural hazards globally [1], are anticipated to have escalating negative impacts. This expectation arises from two primary reasons: First, climate change is projected to increase both the frequency and intensity of floods. Second, there is a significant rise in population density and economic assets in flood-prone areas [2]. Climate change has heightened the likelihood of flooding by up to 50 times, with a temperature increase of 1.2 °C causing floods to become up to 50% more intense [3]. Consequently, floods are expected to exacerbate the global burden of disease, elevate morbidity and mortality rates, and amplify social and economic disruption. These compounded effects will impose severe strains on health services, especially in countries with limited resources [4].

Floods, like other disasters, yield both direct and indirect health impacts. Direct effects stem from the physical and primary elements of the disaster, while indirect effects emerge due to the unsafe and unhealthy conditions created by the disaster [5]. In the short term, floods can lead to acute trauma, injuries, toxic exposures, infectious diseases, and water and vector-borne illnesses. Over the long term, they can worsen chronic disease control and adversely impact psychosocial health and nutrition. Postflood outbreaks of infectious diseases present a significant risk, often leading to higher mortality and morbidity rates, especially if left untreated. Chronic disease exacerbations following floods frequently contribute to increased admissions to health facilities. Physical illness and severe mental disorders increase the burden of diseases, especially among vulnerable groups affected by floods [4]. Given their profound short and long-term effects, flood-related health impacts should be thoroughly assessed and presented to decision-makers, alongside essential public health messaging.

By September 2023, Storm Daniel had triggered extensive flooding in northeastern Libya, resulting in widespread damage to homes, health facilities, water networks, and other infrastructure facilities. Damage to health facilities and the loss of healthcare personnel significantly hindered access to medical services [6]. Additionally, this affected area, rich in natural resources, faced extensive environmental destruction, impacting coastal ecosystems, forests, and agricultural lands [3].

The storm and subsequent flooding in Libya were also a climate change disaster. By the time the storm reached the northern coast of Libya, 267 times the long-term average daily rainfall for September had fallen in 24 h. The unexpectedly overwhelming rainfall and the collapse of the Derna dams culminated in a catastrophic event. Derna, one of Libya’s coastal cities, suffered the worst damage as two dams gave way, submerging entire neighborhoods with water levels reaching up to three meters in some areas [3].

The flood disaster triggered by Storm Daniel occurred in densely populated coastal regions of Libya and had a direct impact on areas inhabited by approximately 1.5 million people, corresponding to nearly 22% of the country’s population. [3] Approximately 250,000 people were impacted by the floods in Libya. In the initial phase, 44,800 people were displaced, and over 5000 lost their lives [7]. As of October 2023, over 8000 people remain missing [6]. Including the missing, the total death toll is estimated to be around 15,000, marking it as the second deadliest disaster of 2023 after the earthquake in Türkiye [8]. The financial toll of the floods in Libya stands at approximately United States Dollar (USD) 1.7 billion, equating to about 3.6% of Libya’s gross domestic product in 2022. Recovery and reconstruction costs following the floods are estimated at USD 1.8 billion [3].

While some flood impacts are easily measurable, significant gaps remain in understanding the full spectrum of human consequences. Studies assessing the health impacts of floods are crucial to inform decision-making before, during, and after such events [9]. Epidemiologic investigation of disasters plays a crucial role in disaster preparedness, response, and recovery. Public health surveillance during disasters provides essential information for effective public health interventions. The information obtained through disaster surveillance guides public health actions and enhances disaster planning, research, and training, ultimately reducing the impact of future disasters [5].

This study aims to evaluate the health impacts experienced by individuals seeking care after the Libya flood disaster and to document the clinical responses provided by emergency medical teams, with the goal of contributing to future disaster preparedness and capacity-building efforts.

## 2. Materials and methods

This descriptive study was conducted as part of an international aid initiative following the flood disaster in Libya. Three temporary health clinics were established in Derna, the disaster-affected region, to examine and provide care to individuals seeking medical assistance. The clinics started providing services on September 13, 2023-three days after the flood-and remained operational until November 24, 2023. The study period corresponded to this operational timeframe. The dates of patient visits were recorded in reference to the date of the flood. During this period, a total of 58 specialist/general physicians and 135 allied health personnel provided healthcare services on a rotational basis.

Data on the disaster victims were recorded daily by the examining physician. The data classification was adapted from the Centers for Disease Control and Prevention’s (CDC) Natural Disaster Morbidity Surveillance Individual Form for Active Surveillance with Medical Staff [10].

Disaster victims were categorized by sex, age group, day of visit, and reason for visit. Sex was classified as male or female; age was categorized into 0–17, 18–64, and ≥ 65 years; and day of visit was divided into 0–3, 4–30, and >30 days postdisaster. Reasons for visit were grouped into five main categories: Acute illness/symptoms, exacerbation of chronic disease, injury, mental health, and routine/follow-up. Four of these categories were further divided into subcategories:

Acute illness/symptoms: Conjunctivitis/eye irritation, dermatologic/skin, fever, gastrointestinal, neurological, pain, respiratory, and others.

Exacerbation of chronic disease: Cardiovascular, diabetes, neurological, respiratory, and others.

Injury: Abrasion/laceration/cut, concussion/head injury, fracture/sprain/strain, bite/sting, burn, heat exposure, fall/slip/trip, poisoning, and others.

Routine/follow-up: Medication refill, blood sugar check, blood pressure check, wound care, and others.

Mental health was not subdivided and was analyzed as a single category. Patients whose conditions did not fit any predefined subcategories were classified under ‘Others’ in their respective main categories.

Inclusion criteria were patients with complete and accurate information regarding sex, age, day of visit, and reason for visit. Exclusion criteria were patients with missing or inaccurate data in these areas.

Disaster victims’ data were recorded daily in a Microsoft Excel table and subsequently transferred to IBM SPSS version 27 for statistical analysis. Descriptive statistics were presented using count, percentage, mean, and standard deviation. We used the Chi-Square test to assess categorical variables.

Ethical approval (No: 10/1034) and institutional permission (No: E-72026553-929-263377044) were obtained for the study.

## 3. Results

Between September 13 and November 24, 2023, a total of 5937 records were collected. Of these, 151 were excluded due to missing data. Consequently, a total of 5786 visits by disaster victims were recorded at our clinics during this period. Among these, 362 individuals accounted for 713 repeat visits. Information regarding demographic characteristics is provided, as shown in Table 1: Distribution of visits by sex, age, and day of visit, 75.3% of the disaster victims presenting to the clinic were male, while 24.7% were female. In terms of age, individuals aged between 18 and 64 years accounted for the highest proportion (77.0%), followed by those aged 0–17 years (15.2%) and those aged 65 years or older (7.8%). With regard to the period subsequent to the visit, the majority of cases occurred between 4 and 30 days (69.8%), while visits made after the 30th day accounted for 29.7% of the total. The least frequent category was visits within the first 3 days, representing only 0.5% of the total.

Details regarding the reasons for visits were provided, as shown in Table 2: Distribution of visit reasons by categories and subcategories. When the reasons for visits were analyzed, the most common reason was acute illness/symptoms (55.8%). This was followed by injury (22.0%), exacerbation of chronic disease (13.9%), routine/follow-up (7.9%), and mental health (0.4%).

When the subcategories within the reasons for visits were examined, the most common cause of injury was abrasion/laceration/cut (18.8%), followed by fracture/sprain (1.5%). In the acute illness/symptoms category, the most frequent reason for visits was pain (18.7%), followed by respiratory issues (17.7%) and gastrointestinal problems (7.3%). In the exacerbation of chronic disease category, cardiovascular conditions (6.9%) topped the list, with respiratory issues (2.2%) and diabetes (1.0%) following. Finally, for routine/follow-up visits, the primary reason was wound care (3.8%), followed by medication refills (0.9%).

Information regarding the distribution of reasons for visit by age groups and sex, based on visit counts, was provided, as shown in Table 3. When reason for visits were evaluated by age groups, the 0–17 age group was most commonly affected by acute illness/symptoms (53.1%), followed by injury (33.0%) and exacerbation of chronic disease (7.3%), routine/follow-up (6.3%) and mental health (0.3%). The 18–64 age group was also primarily affected by acute illness/symptoms (56.8%), followed by both injury (20.8%), exacerbation of chronic disease (14.0%), routine/follow-up (8.0%), and mental health (0.4%). Among individuals aged ≥65 years, acute illness/symptoms remained the most common reason for visits (52.2%) as in other groups; however, exacerbation of chronic disease ranked second (26.1%), differing from other groups. This was followed by injury (12.0%), routine/follow-up (9.7%). No cases of mental health were recorded in this age group. Acute illness/symptoms were most prevalent reasons for visits among men (54.3%) and women (61.0%). Among men, this was followed by injury (26.2%), exacerbation of chronic disease (10.6%), routine/follow-up visits (8.9%), and mental health concerns (0.2%). Among women, exacerbation of chronic disease ranked second (23.9%), followed by injury (9.0%), routine/follow-up visits (5.0%), and mental health (1.1%). There was a statistical difference in the reasons for visits based on age and sex (p < 0.001, p < 0.001). Statistical differences were observed among all subgroups (p < 0.001 for each comparison).

Information regarding the distribution of age groups and sex by day of visit, based on visit counts, was provided, as shown in Table 4. When the day of visit was compared by sex and age group, the majority of visits within the first 0–3 days were made by men (90.3%) compared to women (9.7%). Among age groups, the majority of visits during this period were made by individuals aged 18–64 years (87.1%), followed by 0–17 (9.7%) and ≥65 (3.2%). During 4–30 day period, men again accounted for the majority of visits (79.2%), followed by women (20.8%). In terms of age groups, the 18–64 age group constituted the majority of cases (79.9%), followed by 0–17 (13.1%) and ≥65 (7.0%) age groups. In the > 30 days’ category, men continued to be the majority (66.0%) compared to women (34.0%). In this category, the majority of visits were made by individuals aged 18–64 years (70.0%), followed by those aged 0–17 (20.2%) and ≥ 65 years (9.8%). There was a statistical difference in the visit time based on sex and age groups (p < 0.001, p < 0.001). Statistical differences were observed among all subgroups (p < 0.001 for each comparison). In subgroup analysis, no statistical difference was observed between the 0–17 and ≥ 65 age groups (p = 0.652), whereas all other age group comparisons revealed differences (p < 0.001).

Information regarding the distribution of reasons for visits by day of visit, based on visit counts, was provided, as shown in Figure. When the reasons for visits were analyzed by day of visit, during the first 0–3 days, injury cases were the most frequent (51.6%), followed by acute illness/symptoms (45.2%) and exacerbation of chronic disease (3.2%), with no cases reported for mental health or routine/follow-up visits. For visits between days 4–30, acute illness/symptoms were predominant (56.8%), followed by injury (22.6%), exacerbation of chronic disease (13.0%), routine/follow-up (7.3%), and mental health (0.4%). In the >30 days’ category, acute illness/symptoms remained the most common reason (53.9%), followed by injury (20.1%), exacerbation of chronic disease (16.2%), routine/follow-up (9.5%), and mental health (0.4%). There was a statistical difference in reason for visit and day of visit (χ^2^; p, 39,558; <0.001). Statistically differences were observed among all subgroups (p < 0.001 for each comparison).

## 4. Discussion

This study examined a total of 5786 health visits between September 13, 2023, and November 24, 2023, following the floods in Derna on September 10, 2023, which resulted from the collapse of two dams due to the impact of Storm Daniel.

The significant sex imbalance observed in this study (75.3% males and 24.7% females) may be explained by sociocultural factors. As highlighted in the study by Lee et al., women face barriers in accessing medical care during emergencies, often due to religious and cultural reasons [11].

In terms of age groups, admissions were predominantly observed in the 18–64 age group across all periods. However, there was a decrease in visits in this group over time, while visits increased in the 0–17 and ≥65 age groups. This distribution mirrors that found in the study by Kubo et al., in which patients aged 18–64 accounted for 65.9% of the total admissions, while those over 65 represented 6.1%, which is consistent with our findings [12].

When examining the reasons for visits by subcategories, the six most common causes were abrasion/laceration/cuts, pain, acute respiratory symptoms, gastrointestinal symptoms, cardiovascular issues, and dermatological/skin symptoms. These six causes accounted for 74.6% of all visits. The study by Kubo et al. identified the five most common causes as minor injuries, acute watery diarrhea, malaria, acute respiratory infections, and skin diseases, which are largely consistent with our findings [12]. The predominance of acute illness/symptoms over injuries as the main cause of visits may be due to the fact that the flood-affected area was largely inaccessible to citizens, thereby limiting their involvement in activities like cleaning and repair. As noted in the study by Taji et al., injuries tend to become more pronounced in the later stages of a disaster, primarily as a result of postdisaster cleanup and repair activities [13]. Additionally, the widespread devastation and difficulties in disaster management likely contributed to unsanitary conditions, leading to an increase in admissions related to acute illnesses and symptoms.

Flood disasters often increase the risk of infectious diseases by causing pollution in the water resources and environment, due to the spread of water contaminated with infectious agents. Hashimoto et al. reported an increase in waterborne diseases and eye infections, such as conjunctivitis, as well as respiratory diseases following floods [14]. Similarly, Manzoor & Adesola highlighted that the monsoon floods in Pakistan led to outbreaks of diarrhea, skin and eye infections, and respiratory diseases, including asthma and chronic obstructive pulmonary disease (COPD) [15]. Kouadio et al. observed that, after several past flood disasters, cases of gastroenteritis increased due to contaminated water sources, water transportation and storage contamination, the use of shared utensils, soap shortages, and contaminated food [16]. The same study identified overcrowding, poor ventilation, malnutrition, inadequate shelter or protection from the cold, low vaccination rates, population displacement, and deteriorating access to health services as factors contributing to increased respiratory infections [16]. In addition, Jang et al. observed a rise in hospital visits for injuries, chronic respiratory diseases, and viral infections in flood-affected cities [17]. Similarly, in our study, we observed an increase in the incidence of waterborne diseases, as well as eye infections such as conjunctivitis, gastroenteritis, and viral infections, following the flood. Additionally, there was a notable rise in cases of respiratory conditions, including asthma and COPD, in the affected regions. These findings are consistent with previous studies that have highlighted the public health challenges associated with flood events.

The sex distribution of reasons for visits is also noteworthy. While injuries and routine/follow-up visits were more common among men, acute illness/symptoms, exacerbation of chronic diseases, and mental health issues were more prevalent among women. The findings from Jang et al. support the observation that men were more likely to be admitted for injuries than women, which aligns with our results [17]. Furthermore, although men were the predominant sex in all visit periods, there was a decrease in visits for males and an increase in female visits over time.

The increase in visits between the 4th and 30th days of the disaster compared to the 0–3 days, and the subsequent decrease after the 30th day, may be attributed to damage to healthcare facilities in the region caused by the disaster and the timing of international aid arrival. As stated in Hashimoto et al., the decline in visits after the 30th day is in line with the application trends observed in previous flood disasters [14]. However, this reduction may also reflect the decreasing number of victims in the affected region, particularly as many may have fled the disaster zone.

Regarding the reasons for visits, injury was the leading cause during the first 0–3 days, although this decreased in the following days. Acute illness/symptoms emerged as the most common reason for visits thereafter. However, the number of visits related to acute illness/symptoms decreased after day 30, compared to the 4–30 day period. In contrast, the increasing number of visits due to exacerbation of chronic diseases can be explained by the complicating effects of disasters on the management of chronic diseases.

One of the key strengths of this study is that it directly assessed disaster victims’ visits to temporary healthcare facilities. However, limitations include the fact that data collection started on the third day after the disaster, thus excluding health applications that occurred during the first days. Other limitations include not accounting for changes in the number of disaster survivors in the region and the potential for generalization issues due to the fact that not all individuals in need of medical assistance sought care at these facilities.

## 5. Conclusion

This study, which evaluates data collected at clinics in Libya during the aftermath of the floods triggered by the collapse of two dams following Storm Daniel, highlights several critical findings. The health impacts of disasters are influenced by numerous factors, including cultural and social aspects. It is noteworthy that abrasion/laceration/cut, pain, acute respiratory symptoms, gastrointestinal symptoms, cardiovascular issues, and dermatologic/skin symptoms represent the majority of health issues among disaster victims. While injury-related cases were prominent in the early stages of the disaster, chronic disease exacerbations became more frequent over time-an important consideration for disaster preparedness. Studies evaluating the health effects of disasters contribute to refining public health practices and improving disaster management strategies. Providing effective medical care in response to the evolving health needs over the course of a disaster is essential to reducing its negative health effects.

## Figures and Tables

**Figure:**
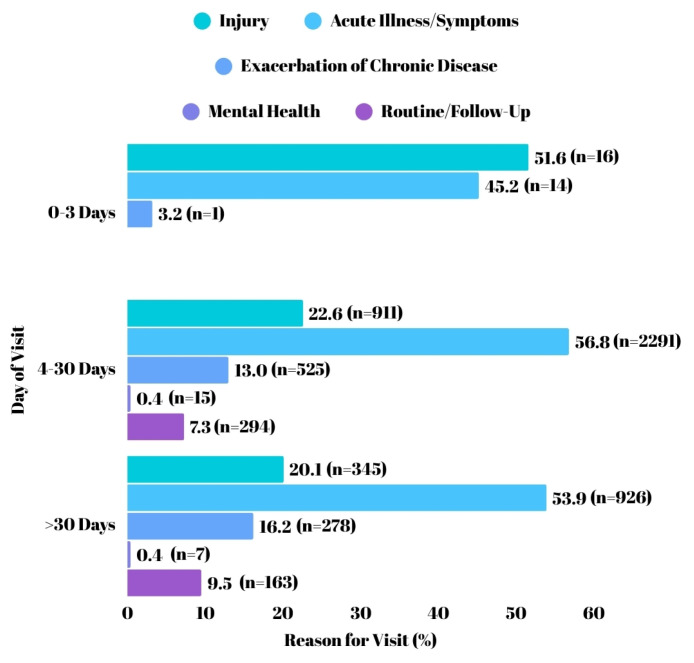
Distribution of reason for visit by day of visit.

**Table 1:** Distribution of visits by sex, age groups, and day of visit.

		N	%
Sex	Men	4358	75.3
	Women	1428	24.7
Age groups (year)	0–17	881	15.2
	18–64	4453	77.0
	≥65	452	7.8
Day of visit	0–3	31	0.5
	4–30	4036	69.8
	>30	1719	29.7
Total		5786	100.0

**Table 2:** Distribution of reason for visit by categories and subcategories.

		N	%
Acute illness/symptoms	Conjunctivitis/eye irritation	118	2.0
	Dermatologic/skin	302	5.2
	Fever	41	0.7
	Gastrointestinal	420	7.3
	Neurological	53	0.9
	Pain	1084	18.7
	Respiratory	1022	17.7
	Others	191	3.3
	Total	3231	55.8
Exacerbation of chronic disease	Cardiovascular	402	6.9
	Diabetes	57	1.0
	Neurological	15	0.3
	Respiratory	128	2.2
	Others	202	3.5
	Total	804	13.9
Injury	Abrasion/laceration/cut	1090	18.8
	Concussion/head injury	14	0.3
	Fracture/sprain/strain	89	1.5
	Bite/sitting	21	0.4
	Burn	15	0.3
	Heat exposure	8	0.1
	Fall/slip/trip	22	0.4
	Poisoning	6	0.1
	Others	7	0.1
	Total	1272	22.0
Mental health	Mental health	22	0.4
Routine/follow-up	Medication refill	54	0.9
	Blood sugar check	13	0.2
	Blood pressure check	46	0.8
	Wound care	220	3.8
	Others	124	2.2
	Total	457	7.9
Total		5786	100.0

**Table 3:** Distribution of reason for visit by age groups and sex (based on visit counts).

	Age groups (year)						Sex				Total	
	0–17		18–64		≥65		Men		Women			
	881		4453		452		4358		1428		5786	
	N	%	N	%	N	%	N	%	N	%	N	%
Injury	291	33.0	927	20.8	54	12.0	1143	26.2	129	9.0	1272	22.0
Acute illness/symptoms	468	53.1	2527	56.8	236	52.2	2360	54.3	871	61.0	3231	55.8
Exacerbation of chronic disease	64	7.3	622	14.0	118	26.1	462	10.6	342	23.9	804	13.9
Mental health	3	0.3	19	0.4	0	0.0	7	0.2	15	1.1	22	0.4
Routine/follow-up	55	6.3	358	8.0	44	9.7	386	8.9	71	5.0	457	7.9
χ^2^; p	158,762; p < 0.001						334,522; p < 0.001					

**Table 4:** Distribution of age groups and sex by day of visit (based on visit counts).

		Day of visit						
		0–3		4–30		>30		
		N	%	N	%	N	%	χ^2^; p
Age groups (year)	0–17	3	9.7	530	13.1	348	20.2	68,975; p < 0.001
	18–64	27	87.1	3223	79.9	1203	70.0	
	≥65	1	3.2	283	7.0	168	9.8	
	Total	31	100.0	4036	100.0	1719	100.0	
Sex	Men	28	90.3	3196	79.2	1134	66.0	117,093; p < 0.001
	Women	3	9.7	840	20.8	585	34.0	
